# Comparative analyses of chloroplast genomes from 22 Lythraceae species: inferences for phylogenetic relationships and genome evolution within Myrtales

**DOI:** 10.1186/s12870-019-1870-3

**Published:** 2019-06-26

**Authors:** Cuihua Gu, Li Ma, Zhiqiang Wu, Kai Chen, Yixiang Wang

**Affiliations:** 1 School of Landscape and Architecture, Zhejiang A&F University, Hangzhou, 311300 China; 2 Department of Biology, Colorado State University, Fort Collins, CO 80523 USA; 3 School of Environment and Resources, Zhejiang A&F University, Hangzhou, 311300 China

**Keywords:** Lythraceae, Chloroplast genome, Phylogenomic, Myrtales

## Abstract

**Background:**

Lythraceae belongs to the order Myrtales, which is part of Archichlamydeae. The family has 31 genera containing approximately 620 species of herbs, shrubs and trees. Of these 31 genera, five large genera each possess 35 or more species. They are *Lythrum*, with 35; *Rotala*, with 45; *Nesaea*, with 50; *Lagerstroemia*, with 56; and *Cuphea*, with 275 species.

**Results:**

We reported six newly sequenced chloroplast (cp) genomes (*Duabanga grandiflora*, *Trapa natans*, *Lythrum salicaria*, *Lawsonia inermis*, *Woodfordia fruticosa* and *Rotala rotundifolia*) and compared them with 16 other cp genomes of Lythraceae species. The cp genomes of the 22 Lythraceae species ranged in length from 152,049 bp to 160,769 bp. In each Lythraceae species, the cp genome contained 112 genes consisting of 78 protein coding genes, four ribosomal RNAs and 30 transfer RNAs. Furthermore, we detected 211–332 simple sequence repeats (SSRs) in six categories and 7–27 long repeats in four categories. We selected ten divergent hotspots (*ndhF, matK, ycf1, rpl22, rpl32, trnK-rps16, trnR-atpA, rpl32-trnL, trnH-psbA* and *trnG-trnR*) among the 22 Lythraceae species to be potential molecular markers. We constructed phylogenetic trees from 42 Myrtales plants with 8 Geraniales plants as out groups. The relationships among the Myrtales species were effectively distinguished by maximum likelihood (ML), maximum parsimony (MP) and Bayesian inference (BI) trees constructed using 66 protein coding genes. Generally, the 22 Lythraceae species gathered into one clade, which was resolved as sister to the three Onagraceae species. Compared with Melastomataceae and Myrtaceae, Lythraceae and Onagraceae differentiated later within Myrtales.

**Conclusions:**

The study provided ten potential molecular markers as candidate DNA barcodes and contributed cp genome resources within Myrtales for further study.

**Electronic supplementary material:**

The online version of this article (10.1186/s12870-019-1870-3) contains supplementary material, which is available to authorized users.

## Background

Lythraceae belongs to the order Myrtales and is named after the genus *Lythrum* [1]. The flowering family is composed of five subfamilies, Lythroideae, Punicoideae, Sonneratioideae, Duabangoideae and Trapoideae, with 31 genera. The subfamily Punicoideae was formerly the family Punicaceae, and the subfamily Trapoideae was formerly the Trapaceae. The genera *Cuphea*, *Lagerstroemia*, *Nesaea*, *Rotala*, and *Lythrum* represent the largest groups of Lythraceae. Lythraceae species are distributed around the world, with most in tropical regions and some in temperate climate regions [2–7].

Most Lythraceae species are herbs, while shrubs or trees are less common [8]. Lythraceae differ from other plant families by the petals, which are crumpled inside their buds, and the many-layered outer integument of their seeds [2, 3]. Many species occur in aquatic or semiaquatic habitats, such as *Didiplis*, *Rotala*, *Morus* and *Trapa*. Some species in the family are of high economic value, such as *Punica granatum* as a fruit tree, *Trapa natans* as edible food, *Heimia myrtifolia* as an important medicinal plant [9] and *Lawsonia inermis* as a natural dye. Overall, the species of Lythraceae have high economic and ornamental value and are widely used in horticulture [10, 11].

Past studies of Lythraceae have concentrated on morphology [2, 12], palynology [13, 14] and anatomy [15]. However, these studies did not distinguish the intraspecific relationship within Lythraceae. More recently, to deepen our understanding of the relationship among Lythraceae species, the modern branch method was used to make a preliminary estimate of the phylogeny within Lythraceae species [16]. Based on *rbcL* genome data*,* the *psaA-ycf3* spacer in the cp genome and the *ITS* sequence of the nuclear ribosomale DNA, the phylogenetic relationship within Lythraceae ware preliminarily inferred [17]. These two noncoding regions improved the resolution between species in an *rbcL* bifurcation diagram [17]. However, due to the use of certain DNA fragments, these studies lead to incomplete conclusions. Complete cp genomes will provide better solutions to relationship reconstruction within Lythraceae and allow exploration of its phylogenetic position within Myrtales.

The chloroplast is an essential organelle for land plants [18], and is mostly inherited maternally [19]. The cp genome usually consists of a two-stranded DNA molecule, and most cp genomes are 120–220 kb in length with 120–140 coding genes [20, 21]. The cp genome usually has four parts: a large single copy (LSC) region, a small single copy (SSC) region, and two copies of the inverted repeat region (IRA and IRB). Because the cp genome is more conserved and shorter in length than the nuclear and mitochondrial genomes, some cp genome sequence have been used to distinguish species and conduct phylogenetic studies [22–25]. An increasing number of cp genomes have recently been reported because complete cp genome sequences provides better data to distinguish marginal taxa, especially below the species level.

In this study, we report six newly sequenced Lythraceae cp genomes and compare them with those of 16 other species within Lythraceae including nine published cp genomes (*P. granatum*, *H. myrtifolia*, *Lagerstroemia fauriei*, *Lagerstroemia floribunda*, *Lagerstroemia guilinensis*, *Lagerstroemia indica*, *Lagerstroemia speciosa*, *Lagerstroemia subcostata* and *Lagerstroemia intermedia*) downloaded from GenBank and seven unpublished *Lagerstroemia* cp genomes (*Lagerstroemia excelsa*, *Lagerstroemia limii*, *Lagerstroemia villosa*, *Lagerstroemia siamica*, *Lagerstroemia tomentosa*, *Lagerstroemia venusta* and *Lagerstroemia calyculata*). Our objectives were as follows: (1) To detect differences between the cp genomes of 22 Lythraceae species; (2) to select 10 highly variable regions to act as candidate barcodes for identifying related species of Lythraceae; (3) to reconstruct phylogenetic relationships to verify branch relationships within Lythraceae and explore its status in Myrtales.

## Results

### Chloroplast genome structure and content

**Fig. 1:**
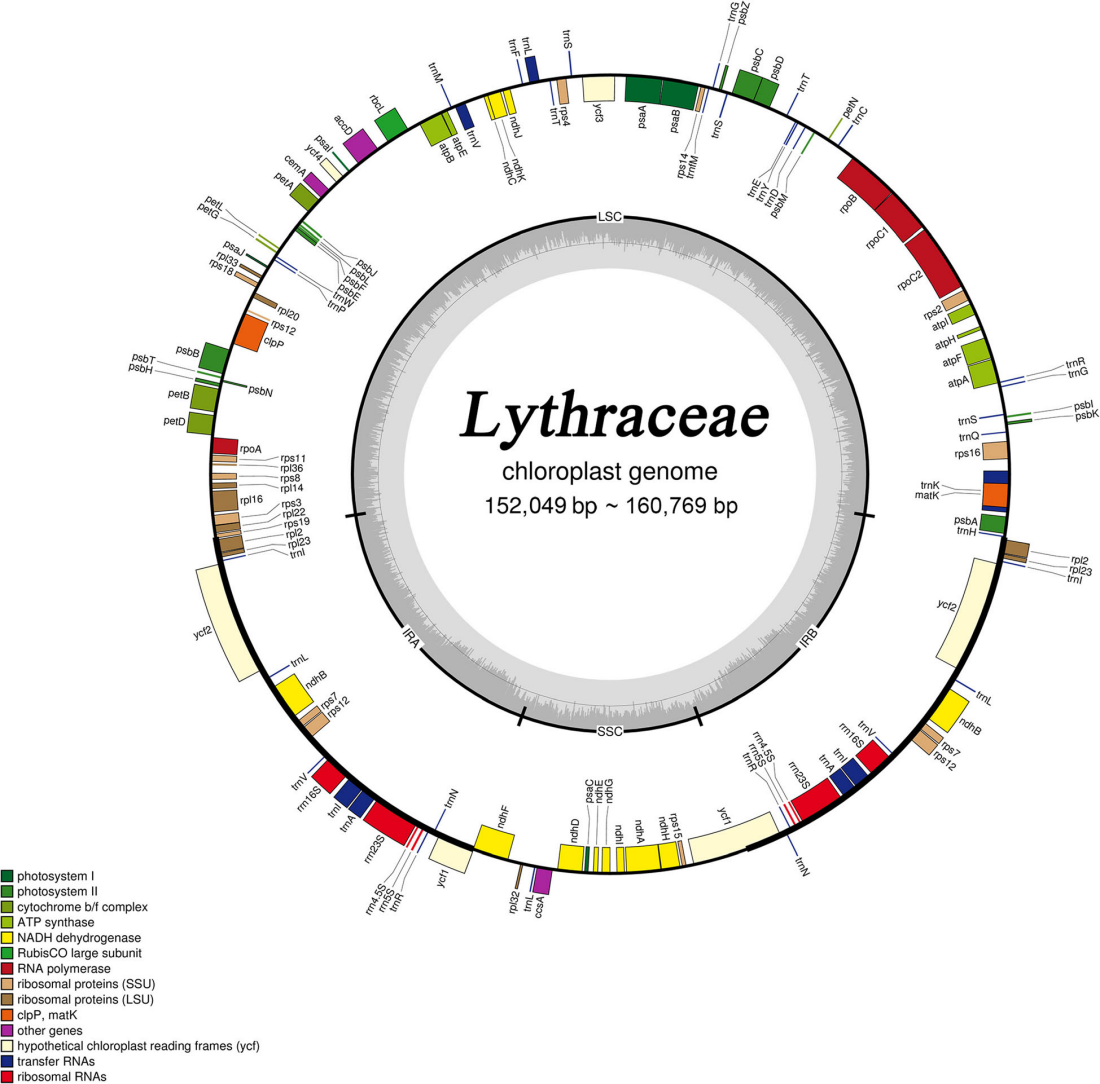
Structural map of the Lythraceae chloroplast genome. Genes drawn inside the circle are transcribed clockwise, and those outside are counterclockwise. Small single copy (SSC), Large single copy (LSC), and inverted repeats (IRa, IRb) are indicated. Genes belonging to different functional groups are color-coded

The complete cp genomes of the 22 Lythraceae species ranged in length from 152,049 bp (*L. subcostata*) to 160,769 bp (*L. villosa*) (Table 1). All cp genomes had the typical four conjoined structures, including the LSC and SSC regions separated by two IR regions (Fig. 1). The LSC regions ranged from 83,817 bp (*L. guilinensis*) to 89,569 bp (*W. fruticosa*) and accounted for 55.10–56.90% of the total length. The SSC regions varied between 16,501 bp (*D. grandiflora*) and 33,301 bp (*L. speciosa*) and accounted for 10.60–21.80% of the total length. The IR regions ranged from 17,541 bp (*L. floribunda*) to 26,906 bp (*L. villosa*) and accounted for 11.50–17.00% of the total length.

**Table 1:** Summary of complete chloroplast genomes for 22 species in Lythraceae

		*L.excelsa*	*L.limii*	*L.villosa*	*L.siamica*	*L.tomentosa*	*L.venusta*	*L.calyculata*	*L.fauriei*	*L.floribunda*	*L.guilinensis*
Accession number		MK881635	MK881627	MK881633	MK881628	MK881632	MK881630	MK881636	NC_029808	NC_031825	NC_029885
Family		Lythraceae	Lythraceae	Lythraceae	Lythraceae	Lythraceae	Lythraceae	Lythraceae	Lythraceae	Lythraceae	Lythraceae
Total length (bp)		152,214	152,153	160,769	152,519	152,294	152,521	152,294	152,440	152,240	152,193
GC(%)		37.6	37.58	36.97	37.58	37.65	37.57	37.65	37.61	37.72	37.62
LSC											
Length (bp)		84,053	83,954	88,702	84,166	84,013	84,194	84,012	83,926	83,967	83,817
GC(%)		35.94	35.92	34.69	35.89	35.98	35.87	35.97	35.94	36.1	35.95
length(%)		55.2	55.2	55.2	55.2	55.2	55.2	55.2	55.1	55.2	55.1
SSC											
Length (bp)		16,917	16,905	18,255	16,865	16,917	16,833	16,798	16,934	16,787	16,909
GC(%)		31.03	30.96	30.78	30.95	31.03	30.97	31.17	30.92	31.23	30.97
length(%)		11.1	11.1	11.4	11.1	11.1	11.0	11.0	11.1	11.0	11.1
IR											
Length (bp)		25,622	25,647	26,906	25,744	25,622	25,747	25,742	25,790	25,788	25,794
GC(%)		42.49	42.47	42.83	42.51	42.49	42.51	42.51	42.51	42.48	42.47
length(%)		16.8	16.9	16.7	16.9	16.8	16.9	16.9	16.9	16.9	16.9
*L. indica*	*L.speciosa*	*L.subcostata*	*L.intermedia*	*D.grandiflora*	*T.natans*	*L.salicaria*	*L.intermis*	*P.granatum*	*W.fruticosa*	*R.rotundifolia*	*H.myrtifolia*
NC_030484	NC_031414	NC_034952	NC_034662	MK881638	MK881634	MK881629	MK881631	NC_035240	MK881637	MK881626	MG921615
Lythraceae	Lythraceae	Lythraceae	Lythraceae	Lythraceae	Lythraceae	Lythraceae	Lythraceae	Lythraceae	Lythraceae	Lythraceae	Lythraceae
152,205	152,476	152,049	152,330	156,084	155,555	158,483	157,756	158,639	159,380	157,753	159,219
37.59	37.58	37.59	37.59	37.49	36.41	36.81	36.89	36.92	36.63	36.89	37.00
LSC											
84,046	84,051	83,890	83,987	86,471	88,506	88,999	88,424	89,022	89,569	88,422	88,571
35.93	35.89	35.92	35.92	35.59	34.19	34.75	34.76	34.89	34.53	34.76	35.00
55.2	55.1	55.2	55.1	55.4	56.9	56.2	56.1	56.1	56.2	56.1	55.6
SSC											
16,915	16,886	16,909	16,871	16,501	18,274	18,530	17,386	18,685	18,697	17,386	18,822
30.98	30.97	30.97	30.93	31.28	30.18	30.68	31.01	30.63	30.23	31.01	30.60
11.1	11.0	11.1	11.1	10.6	11.7	11.7	11.0	11.8	11.7	11.0	11.8
IR											
25,622	25,817	25,625	25,736	26,556	24,388	25,477	25,973	25,466	25,557	25,973	25,643
42.5	42.51	42.5	42.51	42.5	42.77	42.63	42.5	42.78	42.65	42.5	42.60
16.8	16.9	16.9	16.9	17.0	15.7	16.1	16.5	16.1	16.0	16.5	16.1

*GC* guanine-cytosine, *LSC* large single-copy region, *SSC* short single-copy region, *IRs* inverted repeats

A total of 112 unique genes were detected in the cp genomes of the 22 Lythraceae species, including 78 coding genes, 30 tRNA genes and 4 rRNA genes (Table 2). Among the 22 Lythraceae species, the lengths of the protein coding exons ranged from 73,401 bp (*L. indica*) to 81,047 bp (*H. myrtifolia*), rRNA ranged from 9022 bp (*T. natans*) to 9068 bp (*L.fauriei*), tRNA ranged from 2741 bp (*L. guilinensis*) to 2913 bp (*L. excelsa*), intergenic regions ranged from 44,031 bp (*L. guilinensis*) to 51,367 bp (*L. villosa*) and intronic regions ranged from 14,786 bp (*L. calyculata*) to 18,099 bp (*L. villosa*). Each of these accounted for 37.00–38.00%, 3.00–6.00%, 1.80–1.90%, 28.90–32.40% and 9.70–11.30% of the total length, respectively (Table 3).

**Table 2:** Genes contained in the sequenced Lythraceae chloroplast genome

Gene category	Groups of genes	Name of genes
Self-replication	Ribosomal RNAs	*rrn16* ^*b*^ *;rrn23* ^*b*^ *;rrn4.5* ^*b*^ *;rrn5* ^*b*^
	Transfer RNAs	*trnA-UGC* ^*a,b*^ *;trnC-GCA;trnD-GUC;trnE-UUC;trnF-GAA;trnfM-CAU*
		*trnG-UCC* ^*a*^ *;trnG-GCC;trnH-GUG;trnI-CAU* ^*b*^ *;trnI-GAU* ^*a,b*^ *;trnK-UUU* ^*a*^
		*trnL-CAA* ^*b*^ *;trnL-UAA* ^*a;*^ *trnL-UAG;trnM-CAU;trnN-GUU* ^*b*^ *;trnP-UGG*
		*trnQ-UUG;trnR-ACG* ^*b*^ *;trnR-UCU;trnS-GCU;trnS-GGA;trnS-UGA*
		*trnT-GGU;trnT-UGU;trnV-UAC* ^*a;*^ *trnW-CCA;trnY-GUA*
	Small subunit of ribosome	*rps2;rps3;rps4;rps7* ^*b*^ *;rps8;rps11;rps12* ^*a,b*^ *;rps14;rps15;rps16* ^*a*^ *;rps18;rps19*
	Large subunit of ribosome	*rpl2* ^*a,b*^ *;rpl14;rpl16* ^*a*^ *;rpl20;rpl23* ^*b*^ *;rpl32;rpl33;rpl36*
	DNA dependent RNA polymerase	*rpoA;rpoB;rpoC1* ^*a*^ *;rpoC2*
Photosynthesis	Subunits of photosystem I	*psaA;psaB;psaC;psaI;psaJ*
	Subunits of photosystem II	*psbA;psbB;psbC;psbD;psbE;psbF;psbH;psbI;psbJ;psbK;psbL;psbM*
		*psbN; psbT;psbZ*
	Subunits of cytochrome	*petA;petB* ^*a*^ *;petD;petG;petL;petN*
	Subunits of ATP synthase	*atpA;atpB;atpE;atpF* ^*a*^ *;atpH;atpI*
	ATP-dependent protease subunit p gene	*clpP* ^*a*^
	Large subunit of Rubisco	*rbcL*
	Subunits of NADH dehydrogenase	*ndhA* ^*a*^ *;ndhB* ^*a,b*^ *;ndhC;ndhD;ndhE;ndhF;ndhG;ndhH;ndhI;ndhJ;ndhK*
Other genes	Maturase	*matK*
	Envelop membrane protein	*cemA*
	Acetyl-CoAcarboxylase	*accD*
	other	*ccsA;infA*
Genes of unknown function	Conserved open reading frames	*ycf1* ^*b*^ *;ycf2* ^*b*^ *; ycf3* ^*a*^ *; ycf4*

^a^Intron-containing genes

^b^Genes located in the IR regions

**Table 3:** Distribution of genes and Intergenic regions for 22 species in Lythraceae

		***L.excelsa***	*L.limii*	*L.villosa*	*L.siamica*	*L.tomentosa*	*L.venusta*	*L.calyculata*	*L.fauriei*	*L.floribunda*	*L.guilinensis*
Accession number		MK881635	MK881627	MK881633	MK881628	MK881632	MK881630	MK881636	NC_029808	NC_031825	NC_029885
Family		Lythraceae	Lythraceae	Lythraceae	Lythraceae	Lythraceae	Lythraceae	Lythraceae	Lythraceae	Lythraceae	Lythraceae
Protein Coding Genes											
Length (bp)		79,046	79,080	79,648	79,056	79,062	79,059	79,062	79,062	78,852	79,068
GC(%)		37.86	37.84	37.77	37.8	37.87	37.8	37.86	37.84	37.92	37.86
length(%)		52	52	50	52	52	52	52	52	52	52
rRNA											
Length (bp)		9038	9038	9035	9040	9038	9040	9038	9068	9044	9044
GC(%)		55.72	55.72	55.43	55.62	55.68	55.64	55.68	55.67	55.57	55.71
length(%)		6	6	6	6	6	6	6	6	6	6
tRNA											
Length (bp)		2913	2819	2814	2808	2813	2814	2813	2809	2745	2741
GC(%)		52.8	53.35	53.2	53.45	53.47	53.45	53.47	53.15	53.55	53.37
length(%)		2	2	2	2	2	2	2	2	2	2
Intergenic Regions											
Length (bp)		45,482	46,156	51,367	45,619	45,941	45,486	46,033	44,138	45,266	44,031
GC(%)		32.55	32.43	31.3	32.52	32.49	32.54	32.84	32.37	32.54	32.37
length(%)		30	30	32	30	30	30	30	29	30	29
Intron											
Length (bp)		15,334	15,419	18,099	15,607	15,590	15,606	14,786	15,834	15,877	15,596
GC(%)		38.2	38.19	37.82	38.19	38.32	38.18	37.64	38.42	38.7	38.33
length(%)		10	10	11	10	10	10	10	10	10	10
*L. indica*	*L.speciosa*	*L.subcostata*	*L.intermedia*	*D.grandiflora*	*T.natans*	*L.salicaria*	*L.intermis*	*P.granatum*	*W.fruticosa*	*R.rotundifolia*	*H.myrtifolia*
NC_030484	NC_031414	NC_034952	NC_034662	MK881638	MK881634	MK881629	MK881631	NC_035240	MK881637	MK881626	MG921615
Lythraceae	Lythraceae	Lythraceae	Lythraceae	Sonneratiaceae	Trapaceae	Lythraceae	Lythraceae	Punicaceae	Lythraceae	Lythraceae	Lythraceae
Protein Coding Genes											
73,401	79,044	77,139	79,035	78,993	78,848	78,849	79,006	79,029	78,978	79,000	81,047
38.45	37.79	37.76	37.81	37.88	37.27	37.62	37.54	37.63	37.53	37.54	37.00
48	52	51	52	51	51	50	50	50	50	50	51
rRNA											
9050	9046	9042	9046	9040	9022	9038	9038	9038	9038	9038	9050
55.69	55.56	55.68	55.58	55.55	55.51	55.17	55.28	55.26	55.28	55.28	55.00
6	6	6	6	6	6	6	6	6	6	6	6
tRNA											
2817	2742	2828	2807	2903	2812	2813	2812	2817	2819	2812	2817
53.25	53.61	53.39	53.44	52.77	53.24	53.36	53.31	53.28	53.42	53.31	53.00
2	2	2	2	2	2	2	2	2	2	2	2
Intergenic Regions											
44,535	44,313	44,184	45,346	45,923	48,755	50,851	49,417	51,357	50,989	49,441	50,172
32.35	32.45	32.61	32.58	32.36	30.46	31.32	31.38	31.55	30.99	31.62	32.00
29	29	29	30	29	31	32	31	32	32	31	32
Intron											
16,226	15,861	16,201	16,375	15,879	15,564	15,943	15,915	15,928	15,973	15,915	16,133
37.91	38.33	37.87	37.89	38.27	37.14	37.78	37.79	37.9	37.7	37.79	38.00
11	10	11	11	10	10	10	10	10	10	10	10

*GC* guanine-cytosine, *LSC* large single-copy region, *SSC* short single-copy region, *IRs* inverted repeats

Among the 112 distinct genes, a total of 17 genes contained introns. Three genes (*rps12* and *ycf3*) contained two introns, similar to Melastomataceae cp genomes [26]. Fourteen genes contained one intron, including eight coding genes (*rps16*, *rpoC1*, *atpF*, *petB*, *petD*, *ndhB*, *ndhA*, *rpl16*) and 6 tRNA genes (*trnK-UUU*, *trnL-UAA*, *trnV-UAC*, *trnI-GAU*, *trnA-UGC*, *trnG-UCC*). Of the 17 genes containing introns, one gene was distributed in the SSC regions, three genes was distributed in the IR regions and 13 genes in the LSC regions (Additional file 1: Table S1).

### Codon usage

A total of 79 coding genes were used to estimate the codon usage frequency. They were encoded by 25,068 (*L. indica*) to 27,111 (*L. guilinensis*) codons. The termination codons were UGA, UAG and UAA. For the 22 species, the GCU encoded alanine had the highest RSCU value and the UAC encoded tyrosine had the lowest at approximately 0.45. Among most of the 22 Lythraceae species, the AAA encoded lysine had the highest number of occurrences, at more than 1000. This result was also reported in the cp genomes of *H. myrtifolia*, *Aquilaria sinensis*, *Epipremnum aureum* and *Papaver rhoeas* [9, 27–29]. The RSCU results (Table 4, Additional file 2: Table S2) showed that A or T had a higher nucleotide frequency than G or C in the third codon position. It is often the case in terrestrial species that the third codon position prefers A/T over C/G, and the richness of A/ T in the IR regions may be the main reason [30, 31].

**Table 4:** Codon content of 20 amino acid and stop codon of 79 coding genes of 7 species

		*D. grandiflora*	*T.natans*	*L. salicaria*	*L. intermis*	*P. granatum*	*W. fruticosa*	*R. rotundifolia*
Amino acid	Codon				RSCU^a^			
Ala	GCU	1.75	1.78	1.84	1.63	1.80	1.76	1.72
Ala	GCG	0.51	0.44	0.47	0.61	0.46	0.52	0.53
Ala	GCC	0.67	0.64	0.61	0.68	0.63	0.67	0.64
Ala	GCA	1.07	1.13	1.09	1.09	1.11	1.05	1.10
Cys	UGU	1.38	1.43	1.41	1.23	1.43	1.28	1.20
Cys	UGC	0.62	0.57	0.59	0.77	0.57	0.73	0.80
Asp	GAU	1.57	1.56	1.59	1.57	1.59	1.57	1.56
Asp	GAC	0.43	0.44	0.41	0.43	0.41	0.43	0.45
Glu	GAG	0.49	0.49	0.50	0.50	0.48	0.50	0.47
Glu	GAA	1.51	1.52	1.50	1.51	1.52	1.50	1.53
Phe	UUU	1.30	1.25	1.31	1.32	1.30	1.31	1.31
Phe	UUC	0.70	0.75	0.69	0.68	0.70	0.70	0.69
Gly	GGU	1.25	1.32	1.31	1.14	1.27	1.18	1.20
Gly	GGG	0.70	0.71	0.65	0.86	0.66	0.77	0.82
Gly	GGC	0.47	0.41	0.44	0.51	0.46	0.51	0.51
Gly	GGA	1.58	1.56	1.60	1.49	1.61	1.55	1.47
His	CAC	0.50	0.56	0.51	0.51	0.47	0.49	0.55
His	CAU	1.50	1.44	1.49	1.49	1.53	1.51	1.45
Ile	AUU	1.42	1.34	1.44	1.48	1.43	1.45	1.52
Ile	AUA	0.92	1.03	0.91	0.79	0.90	0.90	0.80
Ile	AUC	0.66	0.63	0.65	0.73	0.67	0.66	0.68
Lys	AAA	1.46	1.46	1.46	1.46	1.47	1.44	1.45
Lys	AAG	0.54	0.55	0.54	0.54	0.53	0.56	0.55
Leu	CUA	1.00	1.24	1.02	1.07	1.02	1.05	1.09
Leu	CUC	0.64	0.59	0.64	0.64	0.66	0.69	0.61
Leu	CUG	0.55	0.58	0.54	0.58	0.53	0.51	0.53
Leu	CUU	1.81	1.59	1.80	1.71	1.79	1.75	1.78
Leu	UUA	1.18	1.20	1.18	1.20	1.19	1.18	1.22
Leu	UUG	0.82	0.80	0.82	0.80	0.81	0.82	0.78
Met	AUG	1.00	1.00	1.00	1.00	1.00	1.00	1.00
Asn	AAC	0.46	0.50	0.45	0.56	0.44	0.47	0.56
Asn	AAU	1.54	1.50	1.55	1.44	1.56	1.54	1.44
Pro	CCA	1.21	1.26	1.22	1.21	1.20	1.219	1.23
Pro	CCC	0.84	0.82	0.78	0.84	0.77	0.761	0.84
Pro	CCU	1.49	1.44	1.50	1.39	1.55	1.503	1.41
Pro	CCG	0.46	0.48	0.50	0.57	0.47	0.517	0.53
Gln	CAA	1.54	1.48	1.54	1.50	1.56	1.558	1.51
Gln	CAG	0.46	0.52	0.46	0.50	0.44	0.442	0.49
Arg	AGA	1.44	1.43	1.45	1.35	1.44	1.398	1.38
Arg	AGG	0.56	0.57	0.55	0.65	0.56	0.602	0.62
Arg	CGA	1.60	1.60	1.63	1.62	1.63	1.599	1.68
Arg	CGC	0.43	0.46	0.41	0.41	0.41	0.485	0.42
Arg	CGG	0.50	0.58	0.47	0.70	0.49	0.606	0.67
Arg	CGU	1.47	1.36	1.49	1.27	1.47	1.31	1.23
Ser	AGC	0.54	0.54	0.50	0.67	0.53	0.664	0.69
Ser	AGU	1.46	1.46	1.50	1.33	1.47	1.336	1.31
Ser	UCA	0.91	1.18	0.94	1.23	0.95	0.978	1.24
Ser	UCC	0.99	0.83	0.96	0.80	0.94	0.931	0.80
Ser	UCG	0.54	0.52	0.52	0.59	0.53	0.596	0.61
Ser	UCU	1.56	1.48	1.58	1.37	1.59	1.495	1.35
Thr	ACC	0.82	0.77	0.81	0.91	0.82	0.869	0.89
Thr	ACA	1.16	1.23	1.17	1.20	1.18	1.122	1.23
Thr	ACG	0.49	0.45	0.47	0.54	0.48	0.539	0.54
Thr	ACU	1.53	1.56	1.55	1.35	1.52	1.469	1.33
Val	GUU	1.49	1.39	1.48	1.53	1.48	1.488	1.54
Val	GUG	0.50	0.57	0.53	0.51	0.53	0.549	0.50
Val	GUC	0.55	0.53	0.53	0.62	0.57	0.593	0.60
Val	GUA	1.45	1.51	1.46	1.34	1.42	1.37	1.36
Trp	UGG	1.00	1.00	1.00	1.00	1.00	1	1.00
Tyr	UAC	0.43	0.46	0.43	0.54	0.44	0.483	0.52
Tyr	UAU	1.57	1.54	1.57	1.46	1.56	1.517	1.48
Stop^b^	UGA	0.88	1.21	0.88	1.17	0.89	1.122	1.21
Stop^b^	UAG	0.80	0.76	0.80	0.71	0.81	0.653	0.74
Stop^b^	UAA	1.32	1.03	1.32	1.12	1.30	1.225	1.045

^a^Relative synonymous codon usage; ^b^Stop codon

### Comparative genomic analysis within 22 Lythraceae species

**Fig. 2:**
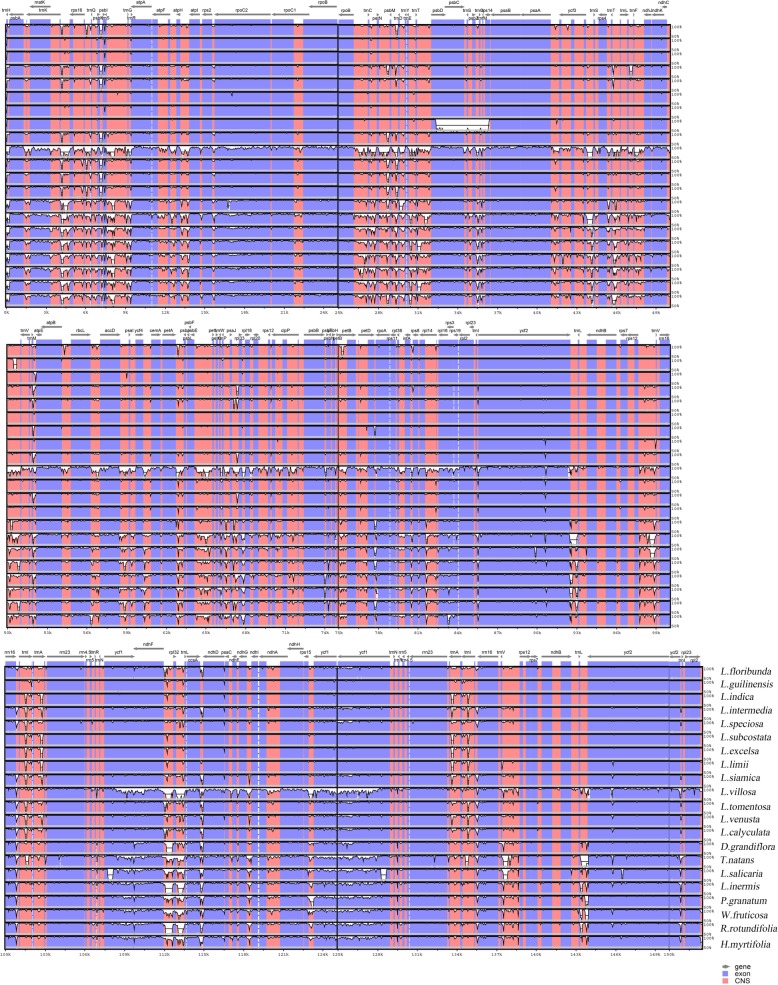
Sequence alignment of whole chloroplast genomes using the Shuffle LAGAN alignment algorithm in mVISTA. *Lagerstroemia fauriei* was chosen to be the reference genome. The vertical scale indicates the percentage of identity, ranging from 50 to 100%

Taking the annotation of *L. excelsa* as a reference, MVISTA was carried out with the cp genome sequences of 22 Lythraceae species. After the 22 cp genomes were pair wise compared, we found that the similarity between the sequences was rather high. From Fig. 2, it is apparent that the 14 *Lagerstroemia* species are separated from the eight other Lythraceae species. The divergence among the 14 *Lagerstroemia* species was low. The LSC and SSC regions had more variation than the IR regions, and the noncoding regions had greater differentiation than the coding regions. Some regions contained more variation, such as *ndhF*, *ndhH*, *matK*, *ycf2*, *rpl22*, *accD*, *rpoB*, *rbcL*, *psbK* among the coding genes and *psbM-trnD*, *trnI-trnA*, *ndhF-rp132*, *rp132-trnL*, *ndhD-psaC*, *atpA-atpF*, *trnI-GAU* intron*, trnK-rps16*, *trnH-psbA* among the intergenic regions (Fig. 2). Similar divergence levels were measured for these regions previously [32, 33].

Compared to the LSC and SSC regions of the 22 cp genomes, the IR regions were most conserved in terms of the sequence and number of genes. However, large variations also existed in connections between the IR, LSC and SSC regions. Inversion and translocation were not detected in the compared genomes. IR amplification and contraction were the main reasons for the difference in the size of these 22 cp genomes.

Significant differences in evolutionary rates were present among the genes across the 22 Lythraceae species analyzed. Overall, the mean Ka/Ks were less than 0.5 for most genes (92.21%). 17 genes showed Ka/Ks higher than 1 for at least one species. Among the 17 genes, seven genes (*rbcL*, *psbJ*, *rpl2*, *rpl20*, *rpl23*, *ccsA* and *ycf4*) presented these high rates for at least 15 species. The results showed that the seven genes may be under positive selection. Seven genes associated with photosynthesis (*psbN*, *psbI*, *psaC*, *atpH*, *petD*, *psbD* and *psbM*) showed the lowest rates of evolution (mean Ka/Ks = 0 to 0.0057), and showed uniform rates in most species evaluated. The Ka/Ks of *psbN*, *psbI*, *psaC* and *atpH* were 0 because there were no non-synonymous substitutions (Additional file 3: Table S3).

In order to detect a possible evolutionary rate acceleration in particular phylogenetic branches, We analyzed three genes with most variable Ka/Ks, namely *rpl23* (large subunit of ribosome), *rbcL* (large subunit of rubisco) and *ycf4* (genes of unknown function). Since the Ka/Ks in comparison among 14 *Lagerstroemia* species were almost 0, we compared the Ka/Ks at *rpl23*, *rbcL* and *ycf4* in comparison of 14 *Lagerstroemia* species and the remaining eight Lythraceae species. For the *rpl23* gene, the Ka/Ks ranged from 0.891 to 1.8077 except for the comparison with *D. grandiflora*. There was no non-synonymous substitution between *Lagerstroemia* species and *D. grandiflora* in addition to *L. excelsa*. As seen in the phylogenetic tree, the relationship between the *D. grandiflora* and the 14 *Lagerstroemia* species was closer than the other seven Lythraceae species. For the *rbcL* gene, the Ka/Ks ranged from 0.1119 to 0.3849, which may be due to a low Ks value (0.0046–0.0177). For the *ycf4* gene, in addition to the comparison with *W. fruticosa* (2.4259–2.8340), the ratio of *Lagerstroemia* species and other seven Lythraceae species ranged from 0.0305 to 0.8758. The result showed that the *rpl23* gene evolved faster than *rbcL* and *ycf4*. The Ka/Ks for the three genes of clade *L. intermis*-*R. rotundifolia* were invalid due to the Ks was 0. The Ka/Ks for the *ycf4* and *rbcL* of clade *P. granatum*-*W. fruticosa* were 0.04 and 2.205, for *rpl23* was invalid (Additional file 3: Table S3).

### Genome size differences among the 22 Lythraceae cp genomes

Of the 22 Lythraceae species, *L. subcostata* was the shortest (152,049 bp), and *L. villosa* was the longest (160,769 bp). Except for *L. villosa*, the lengths of the cp genomes of *Lagerstroemia* species varied between 152,049 bp and 152,519 bp, while the cp genomes of the other genera of Lythraceae varied from 155,555 bp to 159,380 bp (Table 1). In general, the cp genomes of 13 *Lagerstroemia* species were significantly smaller than those of other Lythraceae. The longer length of the cp genome of *L. villosa* resembled those of the 6 newly sequenced species of Lythraceae more than it resembled the *Lagerstroemia* species. The lengths of the intergenic regions (IGS) ranged from 44,031 bp to 46,156 bp among the 13 *Lagerstroemia* species and 45,923 bp to 51,357 bp among the remaining species of Lythraceae, which was in accord with the lengths of the complete cp genomes (Table 4). As in other angiosperm plants, differences in IGS length contributed greatly to the variation in genome size. The percentage of GC content in the chloroplast genomes of the 22 species was 36.41–37.72%, with an average of 37.34%. The average GC content of *Lagerstroemia* species was 37.56%, which was higher than that of the other genera (36.88%).

### Contraction and expansion of inverted repeats (IRs)

**Fig. 3:**
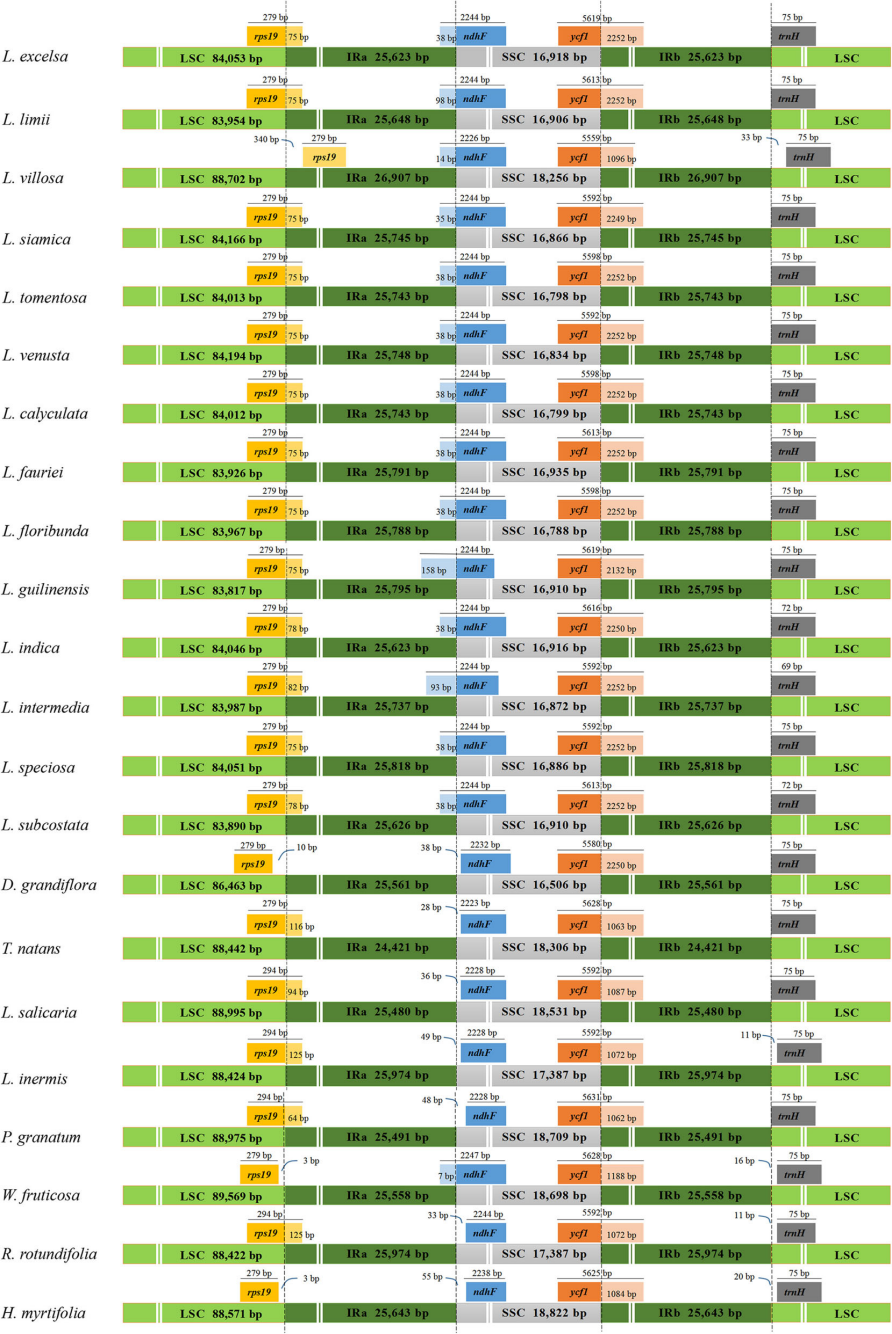
Comparison of junctions between the LSC, SSC, and IR regions among 22 species. Distance in the figure is not to scale. LSC, Large single-copy; SSC, Small single -copy; IR, inverted repeat

The genomic structure, including the number and sequence of genes, was highly conserved among the 22 Lythraceae species. However, there were structural changes in the IRA and IRB boundaries (Fig. 3). Although the IR region is more conserved than the other regions, the enlargement and contraction of IR boundaries played a major role in genome size [34–36].

The sizes of the IRs varied from 24,421 bp (*T. natans*) to 26,907 bp (*L. villosa*). Within the IRA-LSC boundaries of the 22 species, the boundaries of 18 species fell within the *rps19* coding gene and caused an *rps19* pseudogene in the IRB region. The IRA-LSC boundary of *L. villosa* was located on the left side of the *rps19* coding gene and the IRA-LSC borders of *D. grandiflora*, *W. fruticosa* and *H. myrtifolia* were located on the right of the *rps19* coding gene. The distance between *rps19* and the *IRA-LSC* boundary ranged from 3 bp to 279 bp.

Except for the 14 *Lagerstroemia* species and *W. fruticosa*, the IRA-SSC boundary was embedded in the *ndhF* encoding gene and had a length of 7 bp (*W. fruticosa*) to 158 bp (*L. guilinensis*) in the IRA region. For the other 7 Lythraceae species, *ndhF* was located on the right side of the IRA-SSC at a distance of 28 bp to 55 bp from the boundary. For all species, the SSC-IRB boundary was located in the *ycf1* gene with a length of 1062 bp to 2252 bp in the IRB region, causing a *ycf1* pseudogene in the IRA region with a corresponding length. The *trnH-GUG* noncoding gene was located on the right side of the IRB-LSC boundary ranging from 69 bp to 75 bp at a distance of 0 to 33 bp from the IRB-LSC boundary.

### Long repeat structure analysis

**Fig. 4:**
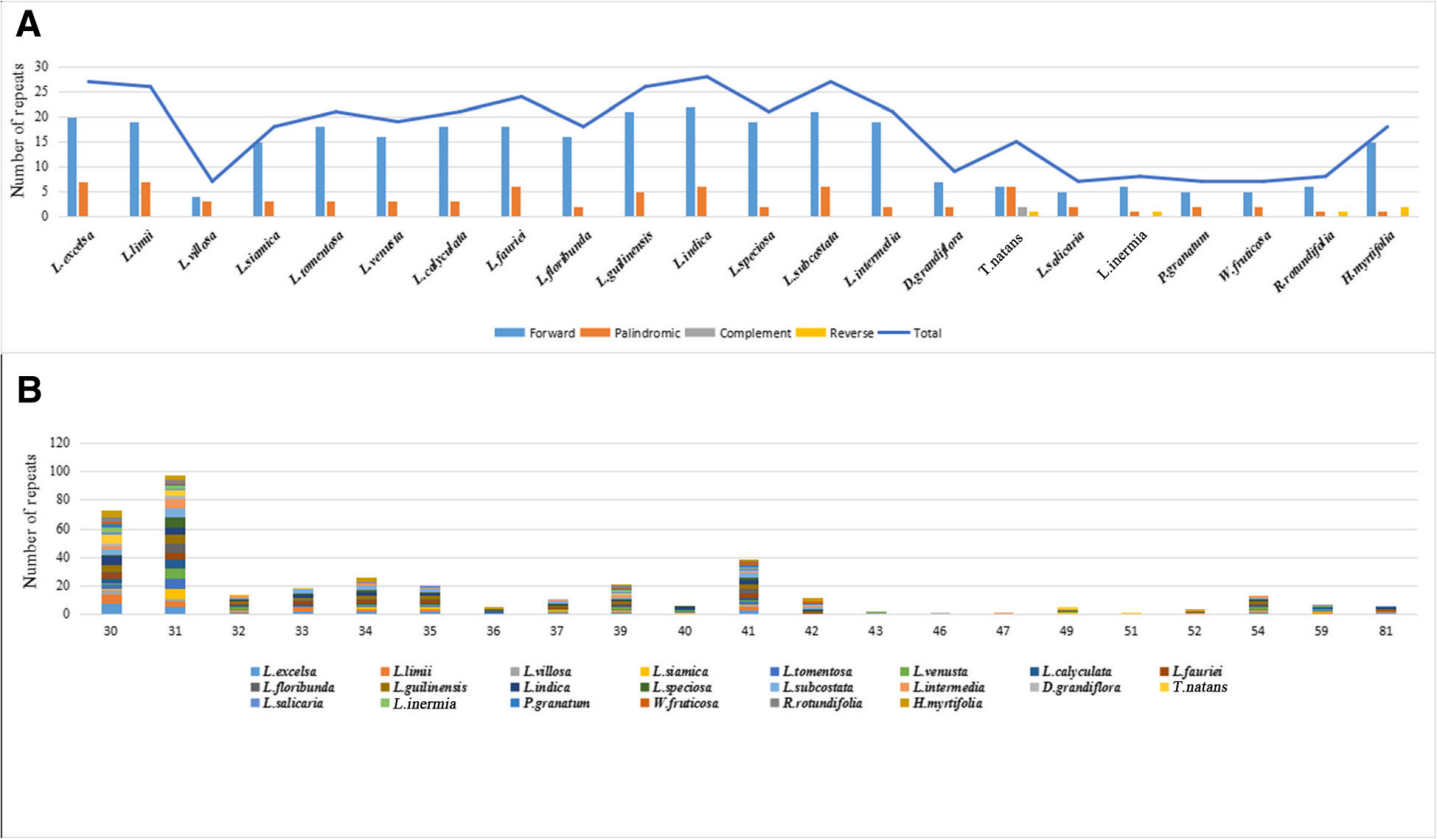
Number of long repetitive repeats on the complete chloroplast genome sequence of 22 Lythraceae species. **a** Frequency of repeat type; **b** Frequency of the repeats more than 30 bp long

Twenty-two Lythraceae species had 383 long repeats of four types. Eighteen species had only forward and palindromic repeats, and only *T. natans* had all four kinds of repeats. *L. indica* had the largest number of repeats, including 22 forward and six palindromic repeats. *W. fruticosa*, *P. granatum*, *L. salicaria* and *P. granatum* had only seven long repeats. As a whole, *H. myrtifolia* and the 14 *Lagerstroemia species had more* long repeats than *D. grandiflora*, *T. natans*, *L. salicaria*, *L. inermis*, *P. granatum*, *W. fruticosa* and *R. rotundifolia* (Fig. 4a, Additional file 4: Table S4)*.* The copy length ranged from 30 bp to 81 bp. Repeat sequences of 30, 31 and 41 accounted for most of the total length (Fig. 4b).

### Simple sequence repeat (SSR) analysis

**Fig. 5:**
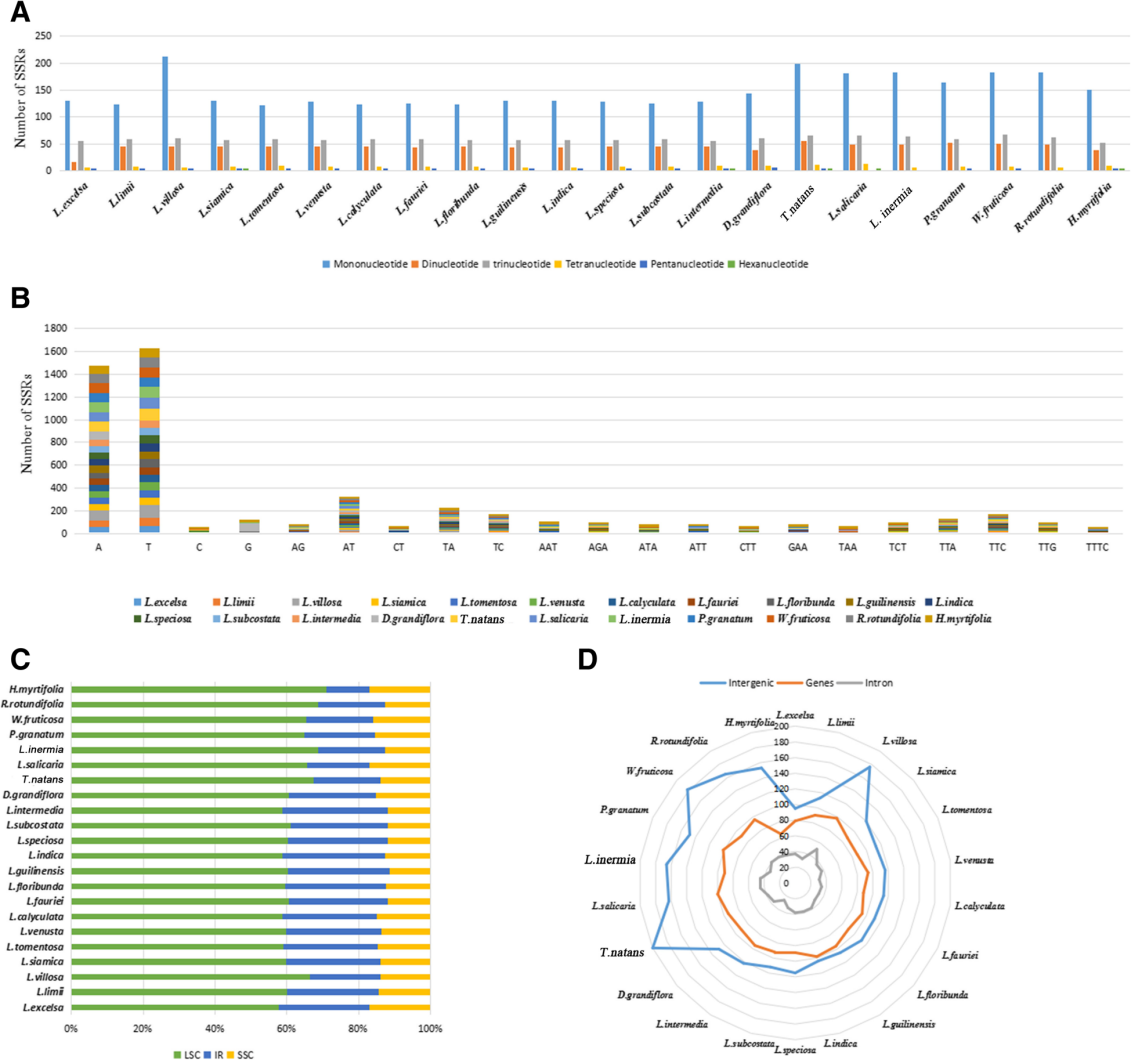
The comparison of simple sequence repeats (SSR) distribution in 22 chloroplast genomes. **a** Number of different SSR types detected in 22 chloroplast genomes; **b** Frequency of common motifs in the 22 chloroplast genomes; **c** Frequency of SSRs in the LSC, IR, SSC region; **d** Frequency of SSRs in the intergenic regions, protein-coding genes and introns

SSRs, also called short tandem repeats or microsatellites, are made up of nucleotide repeat units 1–6 bp in length [37]. SSRs play a significant role in plant taxonomy and are widely applied as molecular markers [38, 39]. There were 211–332 SSRs in each Lythraceae species that ranged from 8 to 16 bp in length (Fig. 5, Additional file 5: Table S5). Six kinds of SSRs were discovered: mononucleotide, di-nucleotide, tri-nucleotide, tetra-nucleotide, penta-nucleotide and hexa-nucleotide. However, hexa-nucleotide repeats were detected in only the cp genomes of *L. siamica*, *L. intermedia*, *T. natans* and *L. salicaria.* Among each Lythraceae species, mononucleotide repeats were the most common, with numbers ranging from 123 to 212; followed by trinucleotide ranging from 56 to 68; dinucleotide ranging from 16 to 52; tetranucleotide ranging from 6 to 12; pentanucleotide ranging from 0 to 2 and hexa-nucleotide ranging from 0 to 1. (Fig. 5a). It was previously found that mono-nucleotide repeats were richest in *Fritillaria*, *Lilium* and *Epimedium* [22, 40]. As a result, mononucleotide repeats may play a more important role in genetic variation than the other SSRs.

In the 22 Lythraceae species, A/T mononucleotide repeats accounted for 45.30 and 50.00%, respectively. C/G mononucleotide repeats accounted for 1.40 and 3.30%, respectively. Most of the other SSRs were composed of A/T, which may have led to the high AT content covering 62.66% of the whole cp genomes within the 22 Lythraceae species (Fig. 5b). Similar biases were also reported in *Quercus* [41]. Moreover, the number of A/T mononucleotide repeats in *D. grandiflora*, *T. natans*, *L. salicaria*, *L. intermis*, *P. granatum*, *W. fruticosa*, *R. rotundifolia* and *H. myrtifolia* were more than 13 *Lagerstroemia* species, ranging from 71 to 92/71–103. Among the 14 *Lagerstroemia* species, the number of A mononucleotide repeats ranged from 54 to 58, with T mononucleotide repeats ranging from 65 to 71, except in *L. villosa*. These results show that the A/T mononucleotide repeats numbers in the same genus are similar. However, the number of A/T mononucleotide repeats of *L. villosa* was 88/117, which was much higher than those of the other 13 *Lagerstroemia* species. We can infer that the longer intergenic spacers are the main reason.

SSRs were much more frequently located in the LSC regions (62.90%) than in the IR regions (23.20%) and the SSC regions (13.90%) (Fig. 5c). Furthermore, SSRs in the cp genomes of the Lythraceae species were located mainly in the intergenic spacers, with an average of 132. SSRs dispersed in coding genes were second, with an average of 92. The fewest SSRs were located in the introns, with an average of 37 (Fig. 5d). The SSR loci were located in 31 coding genes (*matK*, *atpI*, *rpoC2*, *rpoB*, *trnS-UGA*, *rps14*, *psaB*, *psaA*, *ndhK*, *accD*, *ycf4*, *cemA*, *petA, psaJ, psbB*, *rpoA*, *rpl22*, *rps19*, *rpl2*, *ycf2*, *rrn23*, *ndhF*, *rpl32*, *ccsA*, *ndhD*, *ndhA*, *ycf1*, *trnI-GAU*, *ndhB*, *ycf2*) and 57 intergenic regions of the 22 Lythraceae species. Yu et al. found 20 SSRs located in 9 coding genes (*matK*, *rpoC1*, *rpoC2*, *cemA*, *ndhD*, *ndhG*, *ndhH*, *ycf2 and ycf1*) of the *Fritillaria* cp genome [23]. These results indicate that SSRs with large variation in cp genomes can be applied to identify related species and used in research on phylogeny.

### Divergence hotspots among 22 Lythraceae species

**Fig. 6:**
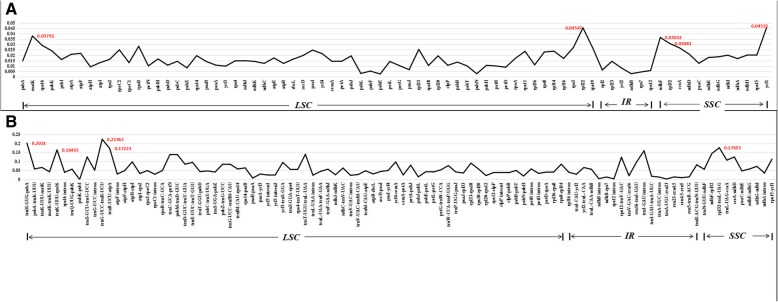
The nucleotide variability (Pi) value in the 22 aligned Lythraceae chloroplast genomes. **a** Protein-coding genes; **b** Intergenic regions

Divergent hotspots on cp genomes can be utilized to identify closely related species and provide information about phylogeny [42, 43]. The nucleotide diversity (Pi) values of the coding regions and intergenic regions of the 22 cp genomes within Lythraceae were computed using the program DnaSP 5.1. It can be seen in Fig. 6 that the values for the intergenic regions were higher than those for the coding regions, indicating that intergenic regions were more differentiated. For the coding regions, the Pi values of the IR region ranged from 0.0029–0.0144, the Pi values of LSC ranged from 0.00261–0.04547 and the Pi values of SSC ranged from 0.01254–0.04532. For the intergenic regions, the Pi value of the IR region ranged from 0.00232–0.15964, the Pi values of the LSC ranged from 0 to 0.22362 and the Pi values of the SSC ranged from 0.03567–0.17653 (Fig. 6, Additional file 6: Table S6). A total of 10 hotspots with high divergence were selected as potential molecular markers to identify related species and examine phylogeny within Myrtales.

Combining the results of DnaSP and mVISTA, we assessed the ability of 10 regions to distinguish the 22 Lythraceae species using ML trees. In the coding regions, the four most variable genes were *ndhF*, *matK*, *rbcL*, and *rpl22*. For the intergenic regions, *trnK-rps16*, *rpl32-trnL, trnM-atpE*, *psbM-trnD*, *trnH-psbA* and *ndhF-rpl32* were the most variable. The regions with the greatest divergence according to their Pi values were similar to the regions obtained from the mVISTA program. Among the 10 divergent hotspots, 7 hotspots were distributed in the LSC region, and the other 3 hotspots were located in the SSC region. The IR regions were so conserved that no highly divergent hotspots were detected. According to the ML trees, *trnK-rps16*, *ndhF*, and *rpl32-trnL* had the highest resolution. The *trnK-rps16* gene clearly separated all the genera within Lythraceae, but the 14 *Lagerstroemia* species could only be divided into five large branches. The *ndhF* gene could also divide all the genera within Lythraceae with bootstrap values of 36–100%, and it separated all 14 *Lagerstroemia* species. Except for the node subtending *L. venusta*, *L. intermedia* and *L. speciosa* with the bootstrap value of 22%*,* the 14 *Lagerstroemia* species were separated with bootstrap values of 64–100%. The *rpl32-trnL* gene divided all the genera except for *Lythrum* and *Heimia*, and the 14 *Lagerstroemia* species could only be divided into five large branches. Compared with *trnK-rps16* and *rpl32-trnL*, *ndhF* had the highest resolution and was the best candidate marker for barcoding.

### Phylogenetic analysis of 22 Lythraceae species with related cp genomes within Myrtales

MP, ML and BI trees were constructed based on the 66 shared protein coding genes of 50 cp genomes (Additional file 7: Table S7). These cp genomes included those of 22 Lythraceae species, 12 Myrtaceae species, three Onagraceae species, five Melastomataceae species and eight species included as out groups. The 22 Lythraceae species included *H. myrtifolia*, *P. granatum*, 14 *Lagerstroemia* species and 6 newly sequenced species (*D. grandiflora*, *T. natans*, *L. inermis*, *R. rotundifolia*, *L. salicaria* and *W. fruticosa*)*.*

**Fig. 7:**
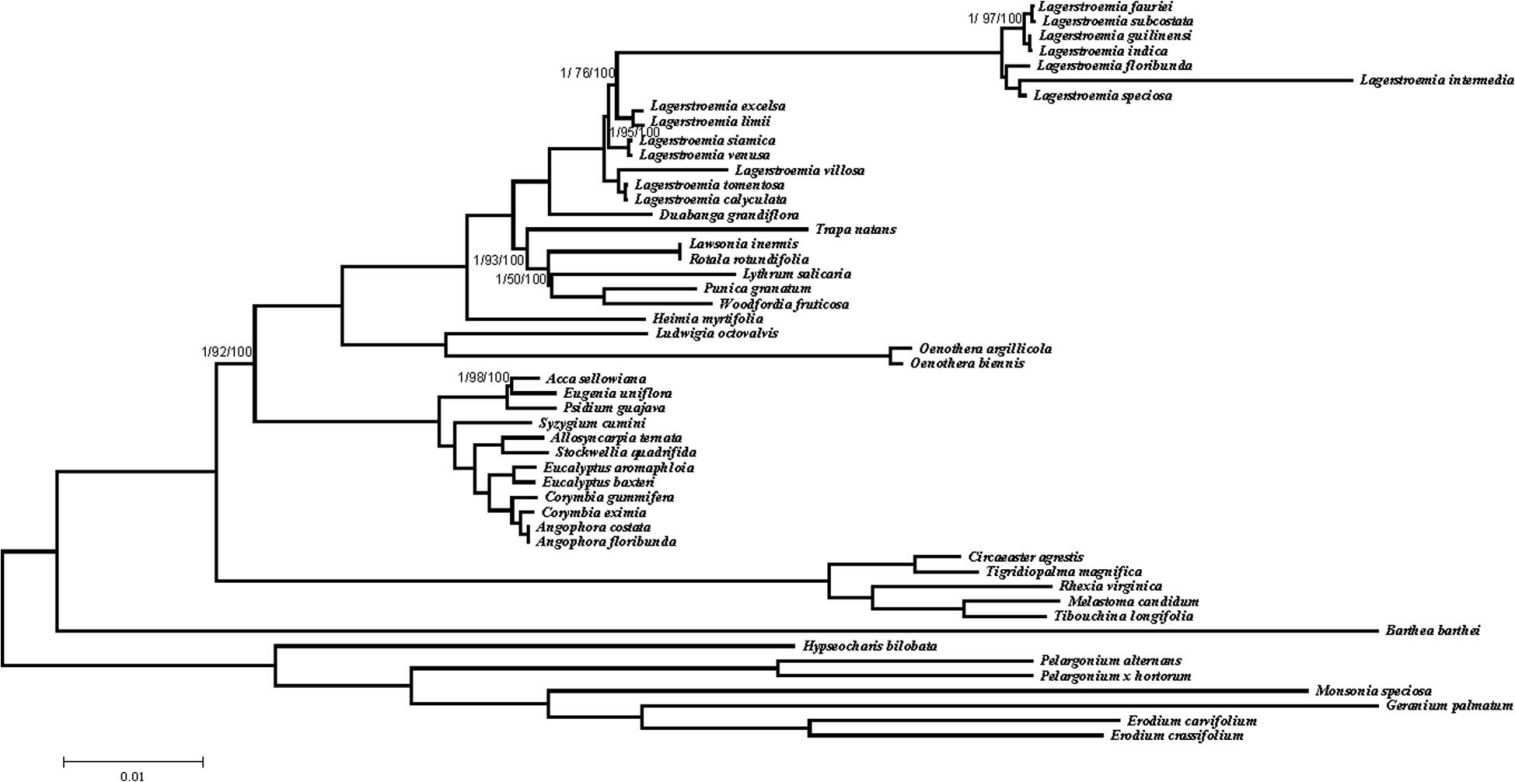
The phylogenetic tree is based on 66 shared protein-coding genes of 50 species. Numbers indicated the bootstrap values from the BI (left), ML (middle) analyses and MP (right) analyses

The topological structures of the ML trees, MP trees and BI trees were consistent, and the four families (Lythraceae, Onagraceae, Myrtaceae and Melastomataceae) were classified into four monophyletic clades. In addition, Melastomataceae was identified as the basal group in Myrtales. The five subfamilies of the Lythraceae gathered into one clade, demonstrating that *P. granatum* and *T. natans*, formerly considered to belong to Punicaceae, and Trapaceae belong to Lythraceae. The 14 *Lagerstroemia* species gathered into one clade. Only two nodes with bootstrap values under 90% in the ML tree. The remaining nodes had support values of more than 92%. The bootstrap values of all nodes reached 100% in the MP tree (Fig. 7). The results showed that the Melastomataceae family, which was sister to the other families within Myrtales, was the earliest differentiating group. The next family to diverge was the Myrtaceae family, followed by the Onagraceae and Lythraceae. The 22 Lythraceae species gathered into one clade, which was resolved as sister to three Onagraceae species (*Ludwigia octovalvis, Oenothera argillicola* and *Oenothera biennis*). As a whole, the phylogenetic tree showed clear internal relationships among Myrtales species.

## Discussion

Each of the 22 Lythraceae cp genomes had four conjoined structures and contained 110–112 distinctive genes consisting of 76–78 coding genes, 29–30 tRNAs and 4 rRNAs. The genome length ranged from 152,049 to 16,0769 bp with GC content between 36.41 and 37.72%. It was clear that the 22 cp genomes were highly conserved in genome size, structure and organization, which were also consistent with the cp genomes of Melastomataceae species reported previously [26]. The largest location of variation among the 22 Lythraceae cp genomes was in the intergenic areas, which is a common phenomenon in cp genomes [10, 44, 45].

The slow evolutionary rate and the low Ka/Ks detected in the analyzed Lythraceae species were within expectations, and Ka/Ks varied among groups of different functional genes. As a common evolutionary pattern for photosynthetic plants, photosynthesis genes (*psbN*, *psbI*, *psaC*, *atpH*, *petD*, *psbD* and *psbM*) had the lowest evolutionary rates. The genes *rpl2*, *rpl20* and *rpl23* involved in replication, *rbcL* and *psbJ* involved in photosynthesis, *ycf4* of unknown functions and other genes including *ccsA* evolved more quickly and had high Ka/Ks (≥1). The seven genes evolved faster among 22 Lythraceae species analyzed were also found in *Capsicum* and *Sesamum indicum* species [23, 46]. Some genes are species-specific in terms of the rates of evolution, such as *clpP* gene. Although it is highly conserved in most green plants, it is by far the fastest evolving plastid-encoded gene in some angiosperms. The rates of evolution in the plastid Clp protease complex are extreme different [47]. The mean Ka/Ks of the *clpP* gene within Lythraceae species was 0.0395, which was different from the high ratio of Ka/Ks in some plants. Williams also found that *clpP1* has undergone remarkably frequent bouts of accelerated sequence evolution, which may result from the intron loss in many lineages, such as *Oenothera*. However, the *clpP* gene contained two introns across 22 Lythraceae species, which may be the reason for its low Ka/Ks. The *clpP* experiencing negative (purifying) selection among Lythraceae species may result from conserved lengths (591 bp). Genes under positive selection typically have large insertions of more or less repeating amino acid sequence motifs [48]. Genes under positive selection may also be bound up with a recent increase in diversification rate after adapted to novel ecological conditions [49].

The boundaries between the four cp genomes regions are important in the evolution of some taxa [50]. For example, pseudogenes such as *ψycf1* or *ψrps19* were produced by contraction and expansion of the IR region. The *ψycf1* pseudogene exists in all 22 Lythraceae species while the *ψrps19* pseudogene was absent in 4 Lythraceae species. The *rps19* gene was located in the LSC regions of *H. myrtifolia*, *W. fruticosa* and *D. grandiflora*. In the cp genome of *L. villosa*, the *rps19* gene was fully duplicated in IRA, as has also been reported in some Malpighiales species [51].

In previous studies, comparative analysis based on complete cp genomes was scarce due to the limited number of published cp genomes of Lythraceae species, and the phylogenetic relationships within Lythraceae were not clear. *P. granatum* and *T. natans* were placed alone in the Punicaceae family and the Trapaceae family respectively. The relationship between *T. natans* and the other species within Myrtales could not be confirmed because of the large morphological variation in *T. natans*, so DNA data were necessary to confirm the location of *T. natans* in Myrtales. The *rbcL* gene, the *pasA-ycf3* spacer, and the *ITS* sequences have been used to establish trees and infer phylogenetic relationships within Lythraceae, and these relationships were corroborated by our results. The sister relationship between *Trapa* and *Sonneratia* was strongly supported, while the sister relationship between *Trapa* and *Lythrum* was weakly supported. Overall, the position of *T. natans* in the family Lythraceae was confirmed in our phylogenetic analysis. Our results further show that *P. granatum* belong to the Lythraceae.

## Conclusion

In this study, the newly sequenced cp genomes of *D. grandiflora*, *T. natans*, *L. salicaria*, *L. inermis*, *W. fruticosa* and *R. rotundifolia* were reported and combined with those of 16 other species to compare a total of 22 Lythraceae cp genomes. The cp genomes of the 22 Lythraceae species were similar in structure, composition and gene order, showing that they are highly conserved. Three phylogenetic trees showed that 42 Myrtales species were completely divided into four branches representing four families with high bootstrap values. From previously existing cp genomes, the evolutionary history of Myrtales had been preliminarily understood. The results of this study provide additional rich genetic resources for phylogenetic research and will play an important role in further study within Myrtales.

## Materials and methods

### DNA extraction of plant materials and sequencing

The fresh leaves of six species of Lythraceae within Myrtales (*D. grandiflora*, *T. natans*, *L. salicaria*, *L. inermis*, *W. fruticosa* and *R. rotundifolia*) were obtained from the nursery of Zhejiang A&F University, and then immediately stored in silica gel. A CTAB method was used to extract the genomic DNA [52]. A NanoDrop 2000 Micro spectrophotometer and an Agilent 2100 Bioanalyzer (Agilent Technologies, Santa Clara, CA) were employed to evaluate the concentration and quality of the extracted DNA. Following the manufacturer’s instructions, the purified DNA was used to build a sequencing library. The Illumina HiSeq 2000 sequencer (Illumina Biotechnology Company, San Diego, CA) was used to obtain paired-end (PE) reads of 150 bp [9].

### Chloroplast genome assembly, annotation, and structure

Trimmomatic v0.3 was used to trim and filter raw reads with a Phred quality score ≤ 20. The other parameters in Trimmomatic v0.3 were set as follows: the sliding window was set to 4:15, the trailing was set to 3, the leading was set to 3 and the minlen was set to 50 [53]. CLC version 9.11 (Qiagen Company, Hilden) with default parameters was used to perform de novo assembly. Four to eight different contigs were created for each species [54]. The BLAST algorithm was used with the *L. fauriei* cp genome as a reference to align all contigs. The ends of each contig could be overlapped by 50 to 80 bp and combined as one large cp genome. The Re-read mapping was also conducted to validate the genome. The coverage of each genome varied from 500x to 900x. DOGMA v1.2 was used to perform genome annotation [8–10, 55]. OGDRAW (http://ogdraw.mpimp-golm.mpg.de/) was used to draw the circular cp genome map of the Lythraceae species and then manually edited [56].

### Codon usage

The relative synonymous codon usage (RSCU) is the ratio of the frequency of the specific codon to the expected frequency [57]. An RSCU > 1.00 means that a codon is used more frequently than expected, while an RSCU < 1.00 denotes that a codon is used less frequently than expected. The RSCU was obtained using DAMBE5 [58].

### Genome comparative analysis and molecular marker identification

A total of 22 Lythraceae species were compared. Taking the *L. excelsa* annotation as the reference, the mVISTA in LAGAN mode was used to make pairwise alignments among the 22 cp Lythraceae species genomes [59].

The 77 protein coding regions of 22 Lythraceae species were used to evaluate evolutionary rate variation. DnaSP 5.1 was to calculate the rates of nonsynonymous (Ka) and synonymous substitutions (Ks) [60]. A total of 13,318 Ka/Ks were obtained; the value could not be calculated if Ks = 0.

MEGA 6 was used to align the cp genomes after manual adjustments in BioEdit software [61]. Then, DnaSP 5.1 was used to separately evaluate the Pi values of the coding and noncoding sequences. Pi values across the complete cp genomes, LSC, SSC, and IR regions were also calculated using DnaSP 5.1 [62].

### Identification of long repetitive sequences and simple sequence repeats (SSRs)

REPuter was used to detect four kinds of long repeats: forward, reverse, palindromic, and complementary repeats [63]. The parameters were set as follows: (1) the minimum repeat was more than 30 bp; (2) the sequence identity was more than 90%; (3) the Hamming distance was equal to 3. Msatcommander 0.8.2.0 was used to detect the location and number of SSRs [64] with the following settings: mononucleotides ≥8; dinucleotides ≥4; trinucleotides, tetranucleotides, pentanucleotide and hexanucleotide SSRs ≥3.

### Phylogenetic analysis

To reconstruct the phylogenetic relationships and examine the phylogenetic status of Lythraceae within Myrtales, the complete cp genomes of 42 Myrtales species were used for analysis. Clustal X 2.1 software with default parameter settings was used to align 66 protein coding gene sequences, with manual adjustments to the alignment ends when necessary [65]. The data matrix used in phylogenetic analysis is provided as supplementary data. Evolutionary relationships were analyzed using MEGA 6 for maximum likelihood (ML) and maximum parsimony (MP), MrBayes 3.1.2 for Bayesian inference (BI) trees [60, 66]. If the bootstrap values of the nodes were equal to 100%, they were not marked on the tree. In all analyses, eight species were considered outgroups. The phylogenetic trees were plotted in FigTree [67].

## Additional files

Additional file 1: **Table S1.** The genes having intron in the 22 Lythraceae chloroplast genomes. (XLSX 50 kb)

Additional file 2: **Table S2.** Codon usage and codon-anticodon recognition pattern of 22 Lythraceae species. (XLSX 117 kb)

Additional file 3: **Table S3.** The rates of Ka、Ks and Ka/Ks of 77 genes among 22 Lythraceae species. (XLSX 1497 kb)

Additional file 4: **Table S4.** The comparison of Long repeats among 22 Lythraceae species. (XLSX 72 kb)

Additional file 5: **Table S5.** The comparison of SSRs among 22 Lythraceae species. (XLSX 525 kb)

Additional file 6: **Table S6.** The nucleotide variability (Pi) value of Protein-coding genes and Intergenic regions. (XLSX 23 kb)

Additional file 7: **Table S7.** The GenBank accession numbers of 50 species using in phylogenetic. (DOCX 17 kb)

## Data Availability

The complete chloroplast genomes of the 13 Lythraceae species have been submitted to the NCBI database under the accession number MK881626 (https://www.ncbi.nlm.nih.gov/nuccore/MK881626), MK881627 (https://www.ncbi.nlm.nih.gov/nuccore/MK881627), MK881628 (https://www.ncbi.nlm.nih.gov/nuccore/MK881628), MK881629 (https://www.ncbi.nlm.nih.gov/nuccore/MK881629), MK881630 (https://www.ncbi.nlm.nih.gov/nuccore/MK881630), MK881631 (https://www.ncbi.nlm.nih.gov/nuccore/MK881631), MK881632 (https://www.ncbi.nlm.nih.gov/nuccore/MK881632), MK881633 (https://www.ncbi.nlm.nih.gov/nuccore/MK881633), MK881634 (https://www.ncbi.nlm.nih.gov/nuccore/MK881634), MK881635 (https://www.ncbi.nlm.nih.gov/nuccore/MK881635), MK881636 (https://www.ncbi.nlm.nih.gov/nuccore/MK881636), MK881637 (https://www.ncbi.nlm.nih.gov/nuccore/MK881637) and MK881638 (https://www.ncbi.nlm.nih.gov/nuccore/MK881638). Other data used in the analysis are included within the article and the additional files.

## Abbreviations

CTAB: Cetyltrimethy lammonium Ammonium Bromide
di-: Dinucleotides
IGS: Intergenic Regions
IRs: Inverted repeats
Ka: Non-synonymous site
Ka/Ks: the ratio of Non-synonymous site and Synonymous site
Ks: Synonymous site
LSC: Large single-copy region
ML: Maximum likelihood
mono-: Mononucleotides
MP: Maximum parsimony
penta-: Pentanucleotides
Pi: Nucleotide diversity values
rRNA: Ribosomal RNAs
RSCU: Relative synonymous codon usage
SSC: Small single-copy region
SSRs: Simple-Sequence Repeats
tetra-: Tetranucleotides
tri-: Trinucleotides
tRNA: Transfer RNAs
