# Supplementary material for: Comparative analyses of chloroplast genomes from 22 Lythraceae species: inferences for phylogenetic relationships and genome evolution within Myrtales
Source: BMC Plant Biol. 2019 Jun 26;19:281. doi: 10.1186/s12870-019-1870-3 (PMC6595698; doi:10.1186/s12870-019-1870-3)
Supplement: Supplementary file 7 — Table S7. The GenBank accession numbers of 50 species using in phylogenetic. (DOCX 17 kb) [file 12870_2019_1870_MOESM7_ESM.docx]

**Additional file 7 The GenBank accession numbers of 50 species using in phylogenetic analysis**

| Number | Species | Genbank number |
| --- | --- | --- |
| 1 | *Acca sellowiana* | KX289887 |
| 2 | *Allosyncarpia ternata* | NC_022413 |
| 3 | *Angophora costata* | NC_022412 |
| 4 | *Angophora floribunda* | NC_022411 |
| 5 | *Corymbia eximia* | NC_022409 |
| 6 | *Corymbia gummifera* | NC_022407 |
| 7 | *Eucalyptus aromaphloia* | NC_022396 |
| 8 | *Eucalyptus baxteri* | NC_022382 |
| 9 | *Eugenia uniflora* | NC_027744 |
| 10 | *Erodium carvifolium* | NC_015083 |
| 11 | *Erodium crassifolium* | NC_025906 |
| 12 | *Geranium palmatum* | NC_014573 |
| 13 | *Heimia myrtifolia* | MG921615 |
| 14 | *Lagerstroemia fauriei* | NC_029808 |
| 15 | *Lagerstroemia floribunda* | NC_031825 |
| 16 | *Lagerstroemia guilinensis* | NC_029885 |
| 17 | *Lagerstroemia indica* | NC_030484 |
| 18 | *Lagerstroemia speciosa* | NC_031414 |
| 19 | *Lagerstroemia subcostata* | NC_034952 |
| 20 | *Lagerstroemia intermedia* | NC_034662 |
| 21 | *Lagerstroemia excelsa* | MK881635 |
| 22 | *Lagerstroemia limii* | MK881627 |
| 23 | *Lagerstroemia villosa* | MK881633 |
| 24 | *Lagerstroemia siamica* | MK881628 |
| 25 | *Lagerstroemia tomentosa* | MK881632 |
| 26 | *Lagerstroemia venusta* | MK881630 |
| 27 | *Lagerstroemia calyculata* | MK881636 |
| 28 | *Duabanga grandiflora* | MK881638 |
| 29 | *Trapa natans* | MK881634 |
| 30 | *Lythrum salicaria* | MK881629 |
| 31 | *Lawsonia inermis* | MK881631 |
| 32 | *Woodfordia fruticosa* | MK881637 |
| 33 | *Rotala rotundifolia* | MK881626 |
| 34 | *Punica granatum* | NC_035240 |
| 35 | *Ludwigia octovalvis* | NC_031385 |
| 36 | *Monsonia speciosa* | NC_014582 |
| 37 | *Oenothera argillicola* | NC_010358 |
| 38 | *Oenothera biennis* | NC_010361 |
| 39 | *Psidium guajava* | NC_033355 |
| 40 | *Pelargonium alternans* | NC_023261 |
| 41 | *Pelargonium x hortorum* | NC_008454 |
| 42 | *Stockwellia quadrifida* | NC_022414 |
| 43 | *Syzygium cumini* | GQ870669 |
| 44 | *Barthea barthei* | NC_035661 |
| 45 | *Melastoma candidum* | NC_034716 |
| 46 | *Rhexia virginica* | NC_031886 |
| 47 | *Tibouchina longifolia* | NC_031889 |
| 48 | *Tigridiopalma magnifica* | NC_036021 |
| 49 | *Circaeaster agrestis* | NC_035872 |
| 50 | *Hypseocharis bilobata* | NC_023260 |
